# Volatile Compound Characterization of Coffee (*Coffea arabica*) Processed at Different Fermentation Times Using SPME–GC–MS

**DOI:** 10.3390/molecules27062004

**Published:** 2022-03-21

**Authors:** Gustavo Galarza, Jorge G. Figueroa

**Affiliations:** Departamento de Química, Universidad Técnica Particular de Loja (UTPL), Calle Marcelino Champagnat s/n, Loja 110107, Ecuador; gxgalarza@utpl.edu.ec

**Keywords:** coffee, volatile compounds, postharvest, SPME, GC–MS

## Abstract

Coffee is a beverage that is consumed due to its flavor and fragrance. In this investigation, we demonstrated the relations between different dry fermentation processes of coffee (aerobic, anaerobic, and atmosphere modified with CO_2_) and fermentation times (0, 24, 48, 72, and 96 h), with pH, acidity, and seven volatile marker compounds of coffee. Volatile compounds were extracted by solid phase microextraction (SPME) and an analysis was performed by gas chromatography–mass spectrometry (GC–MS). A significant effect (*p* < 0.05) between the fermentation time and a decrease in pH was demonstrated, as well as between the fermentation time and increasing acidity (*p* < 0.05). Acetic acid was positively correlated with the fermentation time, unlike 2-methylpyrazine, 2-furanmethanol, 2,6-dimethylpyrazine, and 5-methylfurfural, which were negatively correlated with the fermentation time. The aerobic and anaerobic fermentation treatments obtained high affinity with the seven volatile marker compounds analyzed due to the optimal environment for the development of the microorganisms that acted in this process. In contrast, in the fermentation process in an atmosphere modified with CO_2_, a negative affinity with the seven volatile compounds was evidenced, because this gas inactivated the development of microorganisms and inhibited their activity in the fermentation process.

## 1. Introduction

Coffee is one of the most consumed beverages worldwide [1] due to its flavor and aroma, which are influenced by many factors, including processing methods [2]. Fermentation is a coffee processing method, which is carried out exclusively for mucilage removal. Nevertheless, a relationship between fermentation and the aroma profile has been demonstrated, because a controlled fermentation process contributes to the generation of desirable attributes for coffee quality [3].

Coffee quality depends on physical and sensorial characteristics, such as chemical compound concentrations, which are affected by pectinolytic microorganisms that degrade pulp and mucilage polysaccharides, producing alcohol, acids, and other metabolic compounds, which affect coffee quality [4]. Fermentation can be carried out by three processing methods: dry, wet, and semidry [5]. The process for this investigation was the dry fermentation process. 

The dry fermentation process is characterized as aerobic, which maintains a high concentration of the mucilage’s glucose, fructose, and pectin, promoting microorganism activity on the pulp by enzymes they generate, improving pectinolytic and cellulolytic activities [4]. Some results indicate that coffee beans processed by the dry fermentation method obtain high concentrations of volatile compounds [2]. 

Coffee’s volatile compounds are aldehydes, ketones, alcohol, esters, pyrazines, furans, acids, nitrogen compounds, and phenolic volatile compounds [6]. These compounds are the fundamental components of the coffee aroma profile, which is the most distinguishing characteristic of this beverage, whose concentration can drastically change depending on postharvest methods [6]. Fermentation time increases acids’ volatile compounds [7] but, in general, decreases the concentration of volatile compounds [8], because during a long fermentation time, volatile compound synthesis decreases with the restriction of enzymatic activity, reducing aroma precursors [9], such as pyrazines [10] and furfurans [11].

Volatile compounds are determined by gas chromatography–mass spectrometry (GC–MS), which is a precise analysis of the type and concentration of volatile compounds after an adequate extraction method [8]. For the extraction of these compounds, several techniques have been applied in the food industry for quality control. Nevertheless, solid phase microextraction (SPME) is a simple extraction method, solventless, robust, and replicable, because the fiber accumulates analytes with different types of sorbents, improving coffee volatile compound extraction [12].

Determining the relationship between fermentation and volatile compounds is complicated [3]. The objective of this investigation was to determine the relationship between the fermentation time and the volatile compound concentration of roasted coffee. Moreover, during the fermentation process, three environments were evaluated: aerobic, anaerobic, and an atmosphere modified with carbon dioxide (CO_2_).

## 2. Results and Discussion

### 2.1. Coffee Cherry Moisture

The moisture obtained in the recently harvested coffee cherries was 72.1 ± 0.8%, close to the wet optimal value of 70% in the coffee cherry arabica variety cultivated in Brazil [13].

### 2.2. Dissolved Solids of Coffee Cherries

The soluble solids of the coffee cherries were 19.9 ± 1.1 °Brix, a value within the range of 18–21 °Brix obtained in varieties cultivated and harvested in Brazil [14]. Moreover, the coffee cherry used in this investigation was in the optimal range established of 12–24 °Brix for a coffee adequate for harvesting and processing [15].

### 2.3. Coffee Cherry Color

The coffee cherries obtained L* values of 20.04, because the intensity of luminosity decreases depending on ripening stage [16]. The a* value obtained by coffee cherries was 27.44, a higher value than the 17.90 from the ripened coffee cherries of Carvajal, in 2011, which expressed a high concentration of red shades. In addition, the b* value of the coffee cherries was 9.09, a value close to the range of 11.7–11.81, which determines that coffee is mature [16].

### 2.4. Acidity and pH of Coffee Cherries

The coffee cherry acidity was 1.95 ± 0.2 mL of NaOH at 0.1 N/100 g of coffee, and the pH was 5.7 ± 0.1. The acidity and pH of coffee beans depend on the organic acids generated during the maturation stage [17]. The acidity of Cautaí variety coffee, the same used in this study, was 1.12 ± 0.10 of NaOH at 0.1 N/100 g of coffee [18], values lower than those obtained in this study from the time between harvest and the process, because the coffee cherry was still maturating with the formation of different dissolved solids as sugars and acids [15]. A coffee bean, with an initial pH around 5.0, close to the value obtained in this investigation, is adequate for the fermentation process, because the bean has an optimal environment for microorganism development, which is important in this process [19]. The pH data obtained in this investigation were similar to those of Ribeiro in 2018, who obtained a pH of 5.5, and Jackels in 2005, who obtained a pH of 5.5 to 5.7.

### 2.5. pH of Treated Coffee

**Figure 1:**
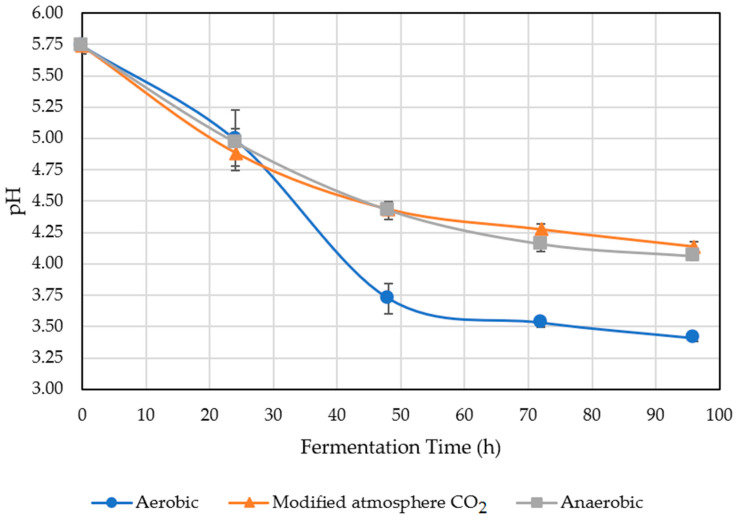
pH of treated coffee.

The decrease in pH was caused by the high population of mesophyll bacteria present in the coffee pulp [20]. Those microorganisms release enzymes that ferment carbohydrates and sugars from the mucilage, causing a pH decrease in the coffee bean [21]. Figure 1 shows the pH obtained per treatment and fermentation time.

In the dry aerobic fermentation process, the decrease in coffee pH was caused by the numerous population of aerobic bacteria that increased their development, permitting sugar consumption and generating a high content of organic acids, such as lactic and acetic, which decreased the pH of the coffee bean [22]. As the fermentation time elapsed, the pH had a tendency to decrease, because after 96 h of fermentation, each treatment obtained the lowest pH value. For that reason, the fermentation time was important, because it affected the population of microorganisms, and it was established that when the coffee bean pH was close to 4.6, there was an adequate time of fermentation [7].

Microorganisms are affected by the pH, which is why the FAO determined that pH, time, and acidity are critical parameters during the fermentation process, because a pH value between 4 and 5.5 has an impact on coffee quality; with a pH under the lower value of 4, the quality of coffee could be affected by overfermentation [23]. Under that condition, the treatments that showed overfermentation were the dry aerobic fermentation after 48, 72, and 96 h. 

Increasing fermentation time restricts the microbial population, maintaining the overfermentation indicators of bacteria, such as lactic acid bacteria tolerant to an acidic environment, which significantly affects coffee quality [19]. Nonetheless, this does not mean that the pH will continue decreasing over time, because it was established that after 64 h, the pH stabilizes [8], as observed in Figure 1.

### 2.6. Acidity of Treated Coffee

Coffee acidity is an indicator of possible changes in the coffee fruit during the fermentation process, occurring before and after fruit harvest [24]. As the observed pH decreased, as described in Section 2.5, during fermentation time, the acidity increased due to the acids generated from the degradation of pectin and the sugars of coffee [7].

**Figure 2:**
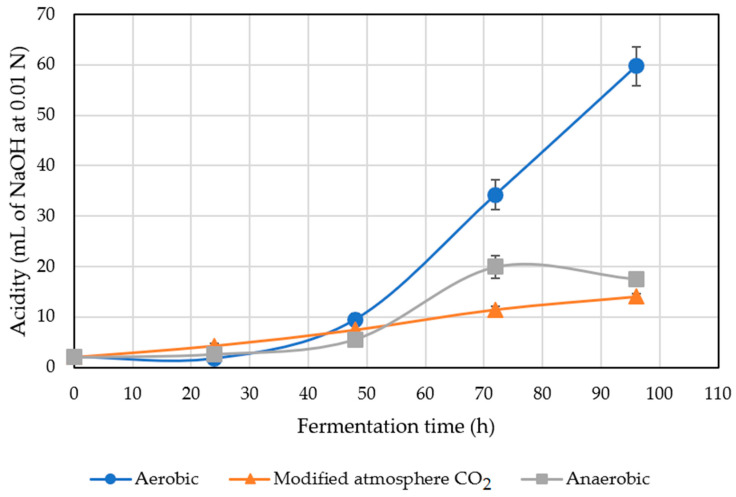
Acidity of the treated coffee.

Mucilage acidification is caused by fermentative bacteria, such as lactic and acetic bacteria, and by the process of alcohol acetification [25]. Latic acid and acetic acid bacteria have a high activity during fermentation of coffee, but they are very susceptible to a CO_2_ environment, because this gas inhibits their development [25]. This can be observed in Figure 2 in the results obtained in the dry fermentation process in an atmosphere modified with CO_2_, where changes in acidity were not high. Dong, in 2019, obtained a high value of acidity in dry aerobic fermentation process in the same conditions as this study, maintaining the tendency to obtain high acidity by the heterogeneous microorganism activities due to the fermentation environment.

### 2.7. Correlation Analysis: Fermentation Time, pH, and Acidity

**Figure 3:**
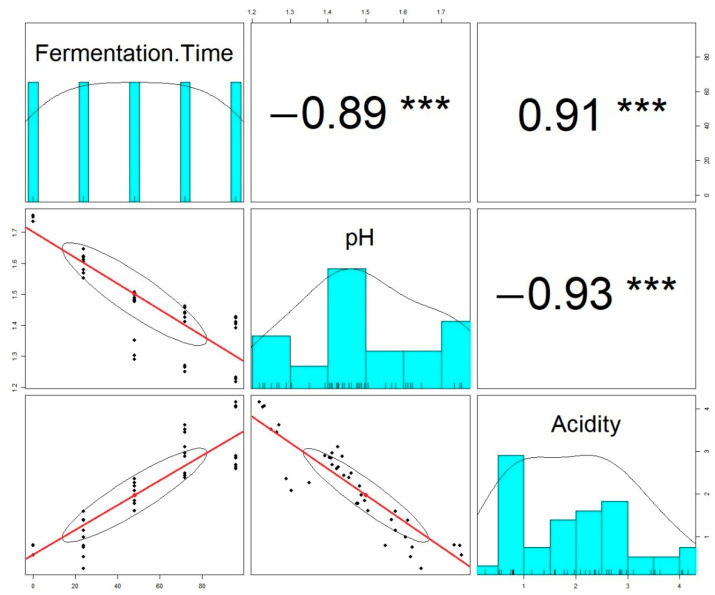
Correlation between fermentation time, pH, and acidity.

The correlation between fermentation time and pH was r = −0.89, which demonstrated that with the increase in fermentation time, the pH decreased. The opposite situation occurred between fermentation time and acidity, whose correlation was r = 0.91, establishing a positive correlation between both parameters. In addition, between pH and acidity, there was a negative correlation of r = −0.93, which showed that both parameters were inversely proportional. In Figure 3, the correlation between the variables was observed, where the symbol *** shows high significance (*p* < 0.05).

### 2.8. Volatile Compounds

Acetic acid (C1), 2-methylpyrazine (C2), 2-furancarboxaldehyde (C3), 2-furanmethanol (C4), 2,6-dimethylpyrazine (C5), 5-methylfurfural (C6), and 2-methoxy-4-vinylphenol (C7) were selected as marker compounds due to their higher abundance. Furthermore, these analytes provided enough information to differentiate arabica and robusta varieties [26]. The identification of volatile marker compounds was performed using the Kovats index (Table 1).

#### 2.8.1. Acetic Acid

**Figure 4:**
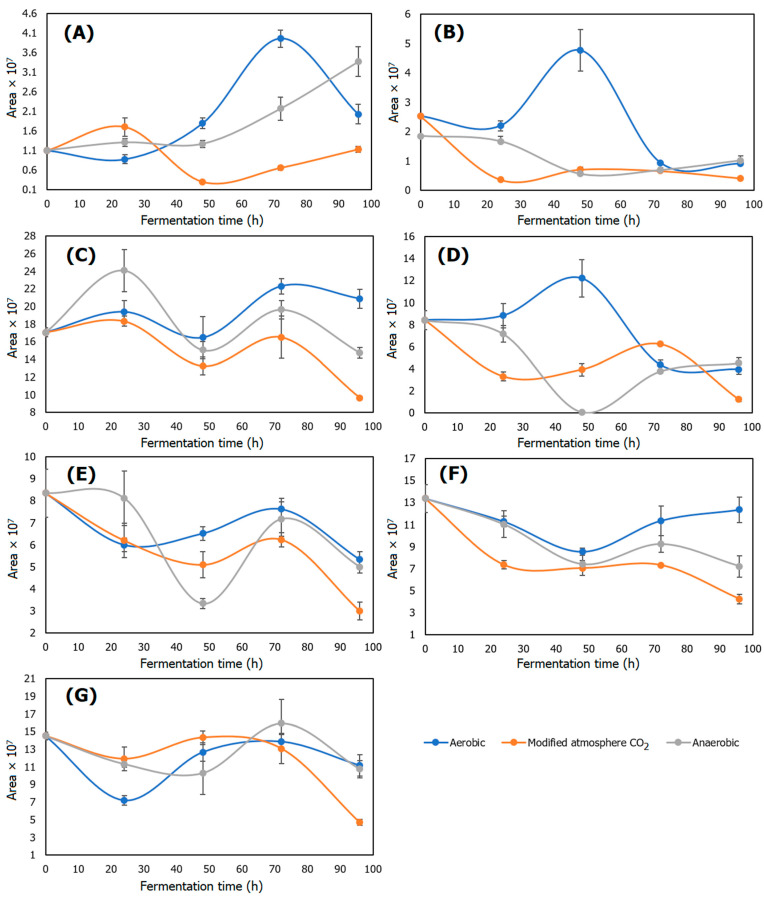
Effect of fermentation time on the concentration of volatile compounds. (**A**) Acetic acid content, (**B**) 2-methylpyrazine content, (**C**) 2-furancarboxaldehyde content, (**D**) 2-furanmethanol content, (**E**) 2,6-dimethylpyrazine content, (**F**) 5-methylfurfural content, and (**G**) 2-methoxy-4-vinylphenol.

The compounds present in a mature coffee cherry are degraded during the fermentation process increasing the formic, lactic, glycolic, and acetic acid content [31]. This occurs because the microorganisms degrade the pulp and mucilage by pectolytic enzyme action, increasing the content of the alcohol and acids, including acetic acid [32]. Acetic acid brings an acceptable flavor of fruit and wine and a fermented aroma [33]. Schwan, in 2012, established that the increment in acetic acid indicates overfermentation, which causes bad coffee quality. Based on that information, Figure 4A shows that the anaerobic and aerobic dry fermentation processes, after 48 h of fermentation, obtained a high content of acetic acid, different to the fermentation in an atmosphere modified with CO_2_, because this gas inhibited acetic acid bacteria developing, as explained in Section 2.6. During aerobic dry fermentation, the acidity increased faster, so it permitted a better consumption of the mucilage substrate and a reduction in acetic acid bacteria due to the acidic conditions of the media [34], decreasing the acetic acid content of the coffee bean by limiting the development of the precursor microorganisms of acetic acid and the consumption of the substrate.

The acetic acid content caused a high decrease in pH in aerobic and anaerobic dry fermentation because the microorganisms developed in optimal conditions [35]. In the dry anaerobic fermentation process, the predominant microorganisms present in the mucilage were lactic acid bacteria *Lactococcus, Leuconostoc,* and *Weissella*, which increased the content of lactic and acetic acid [36]. These bacteria were microaerophile aerobic, which means that they can develop in a low oxygen environment; but in the absence of oxygen, these microorganisms ferment sugars from the media [37], which explains the increase in acetic acid in the dry anaerobic fermentation. Otherwise, in the dry aerobic fermentation process, *Acetobacter* and *Gluconobacter* were predominant due to the aerobic conditions [38]. The metabolic processes of these bacteria, which are part of the acetic acid bacteria, increased the content of the acetic acid at a long fermentation time [39].

#### 2.8.2. 2-Methylpyrazine

2-Methylpyrazine is an indicator compound of coffee cherry quality, which means that when it is present in high concentrations, the coffee is considered high quality [21]. The results in Figure 4B show that the content of 2-methylpyrazine in the dry anaerobic and the atmosphere modified with CO_2_ processes decreased, but it was stable after 24 h. The reason why 2-methylpyrazine, as some pyrazines, did not increase during the fermentation process was because these microorganisms did not have a significant impact in terms of content during the fermentation process [40]. Most pyrazines are synthetized from a Maillard reaction, a process that requires a thermal reaction [12]. However, in the dry aerobic fermentation, there was an increase in 2-methylpyrazine until 48 h of fermentation, because the aerobic fermentation of coffee contains a heterogeneous microorganism population [41], while the anaerobic fermentation process contains a homogeneous population [24]. Thus, the microorganisms present in the aerobic fermentation process, which were more heterogeneous, showed high activity in the coffee mucilage. The reduction in the compound is explained in Section 2.9, caused by the consumption of the substrate and the inactivation of microorganisms. 

#### 2.8.3. 2-Furancarboxaldehyde

The reason that the content of 2-furancarboxaldehyde presented the same behavior between treatments and fermentation times, as observed in Figure 4C, was because the synthesis of furan compounds occurs through the thermal degradation of amino acids and the thermal oxidation of polyunsaturated fatty acids and ascorbic acid [42]; these processes did not occur during fermentation, because it was not a thermal process. Moreover, 2-furancarboxaldehyde is consider as a marker of quality in coffee aroma, bringing a smell of roasted candy [43]. 

#### 2.8.4. 2-Furanmethanol

As observed in Figure 4D, the content of 2-furanmethanol did not have significant changes between fermentation the processes and times, due to the mechanisms of reaction necessary for the synthesis of 2-furanmethanol, such as the Maillard reaction, and other thermal processes, such as oxide thermal degradation of polyunsaturated fatty acids, thiamine, nucleosides, sugars presented during caramelization, carbohydrates, ascorbic acid, and unsaturated fatty acids degradation, processes that require thermal intervention [44]. Moreover, 2-furanmethanol adds roasted aromas, and it is generated by the thermal process that degrades carbohydrates and sugars, such as hexoses and pentoses [45]; but the content of 2-furanmethanol cannot be high, because it generates an undesirable burnt and bitter smell [44].

#### 2.8.5. 2,6-Dimethylpyrazine

2,6-Dimethylpyrazine brings a sweet nutty–fruity aroma [11]. The results in Figure 4E determined that during the fermentation time, the content of 2,6-dimethylpyrazine decreased, because this compound has a tendency to decrease during a fermentation process [12]. At controlled times of fermentation, the content of 2,6-dimethylpyrazine decreased, while acid compounds increased [10], because the interaction between carbohydrates and α-amino acids are necessary for the synthesis of 2,6-dimethylpyrazine [46]; so, as the fermentation time increased, complex carbohydrates were degraded to simple carbohydrates [47], reducing the content of the substrate necessary for 2,6-dimethylpyrazine synthesis.

#### 2.8.6. 5-Methylfurfural

5-Methylfurfural brings sensorial characteristics of candy, spice, and maple syrup [40]. As the fermentation time increased, the content of 5-methylfurfural decreased [11], as observed in Figure 4F. The microbiome present during the fermentation process did not affect 5-methylfurfural synthesis, as it maintained its concentrations between treatments [29]. Evangelista, in 2013, demonstrated that different microorganism strains during fermentation did not affect the content of 5-methylfurfural and showed that there was a significant reduction compared with the initial content of the volatile compounds of coffee. Its reduction in content was, as with 2,6-dimethylpyrazine, due to the reduction in complex carbohydrates caused by microbial activity, which are essential substrates for 5-methylfurfural synthesis [6].

#### 2.8.7. 2-Methoxy-4-Vinylphenol

2-Methoxy-4-vinylphenol, as observed in Figure 4G, maintained its content until 72 h of fermentation time. After 96 h, the content of 2-methoxy-4-vinylphenol decreased due to the acidic environment of the mucilage, which removed or eliminated the methoxy functional group compounds, such as 2-methoxy-4-vinylphenol, reducing its content in coffee beans [27]. Moreover, 2-methoxy-4-vinylphenol is considered beneficial, because it is an indicator of coffee storage quality, whose content increases at storage conditions of 40 °C and 13.5% of moisture [24]. Furthermore, 2-methoxy-4-vinylphenol is characterized as bringing a spicy and floral aroma; however, it is a susceptible compound, because during coffee processing, 2-methoxy-4-vinylphenol has the tendency to decrease [30].

### 2.9. Correlation Analysis: Volatile Compounds, pH, Acidity, and Fermentation Time

As the fermentation time increased, the pH decreased and the acidity increased; there was a highly significant inversely proportional relation between pH and acidity, as shown in Table 2. The correlation of 2-methylpyrazine (C2), 2-furancarboxaldehyde (C3), 2-furanmethanol (C4), 2.6-dimethylpyrazine (C5), 5-methylfurfural (C6), and 2-metoxy-4-vinylphenol (C7) with fermentation time were inversely proportional, in contrast to the acetic acid (C1), which was directly proportional, a tendency reported by Pereira, in 2020, who identified that coffee beans before fermentation processing contained a high content of alcohol and aldehydes, but after fermentation, these compounds decreased, and the content of acids increased. Kim, in 2019, also reported a decrease in volatile compounds in fermented coffee.

### 2.10. Principal Components Analysis (PCA)

**Figure 5:**
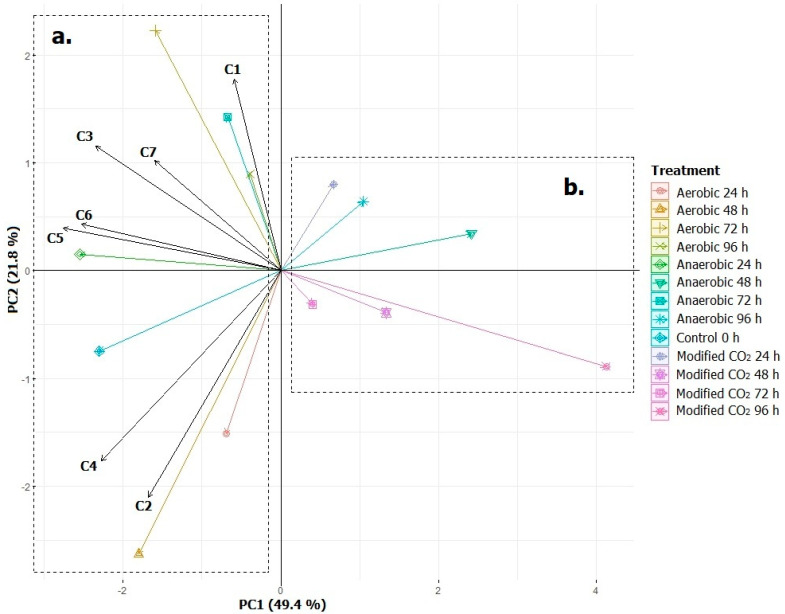
Principal component analysis of each treatment. C1: acetic acid. C2: 2-methylpyrazine. C3: 2-furancarboxaldehyde. C4: 2-furanmethanol. C5: 2,6-dimethylpyrazine. C6: 5-methylfurfural. C7: 2-methoxy-4-vinylphenol.

A PCA analysis was performed to identify the relation between each fermentation process and the concentration of the seven marker volatile compounds. In Figure 5, the PCA is presented, which explained 71.2% of data in the first and second component. There was an affinity between the seven marker volatile compounds with aerobic and anaerobic fermentation treatment (Figure 5a). This is due to the fact that the microorganisms, such as yeast, *Lactobacillus* spp., *Streptococcus* spp., and *Enterobacteriaceae* were anaerobic facultative, which means that they can develop with oxygen. Nevertheless, in an anaerobic environment, these microorganisms fermented the mucilage’s sugars [37]. In another situation, this investigation demonstrated that fermentation in an atmosphere modified with CO_2_ had a negative relation with the seven marker volatile compounds (Figure 5b), due to a decrease in enzymatic activity from microorganism inactivation by the presence of CO_2_ [25]. 

2-Methylpyrazine (C2) showed a better affinity with aerobic fermentation at 24 and 48 h, because there was an inversely proportional relation between this compound and pH [48]. As for acetic acid (C1), its concentration was significantly increased in aerobic fermentation treatments by the heterogeneous aerobic microflora that produced lactic and acetic acid causing a decrease in the coffee mucilage pH, because simple sugars were consumed to produce organic acids [41].

Volatile compounds had a tendency to decrease at 96 h of fermentation, as during the fermentation time, the volatile compounds decreased due to the reduction in precursor substrates and the increase in acid in the fermentation media [10], which explains why the 72 and 96 h aerobic fermentation process and the 96 h anaerobic fermentation process demonstrated a better affinity with acetic acid. 

## 3. Materials and Methods

### 3.1. Coffee Processing

A total of 15 kg of Catuaí variety coffee was obtained from El Aguacate Sauces Norte farm, located in Loja, at 2100 m above sea level. The coffee beans were washed to remove dirt, inert matter, biological matter, and coffee beans that did not meet quality standards. Then, the coffee beans were submerged in water inside metal pots with a recirculating water unit to reduce coffee impurities. After that, 700 g of coffee were weighed using a Mettler Toledo scale, putting each amount of weighed coffee into an autoclavable bottle with a blue thread top (18 in total), each with a capacity of 1000 mL. Each treatment had, in total, 6 bottles. 

### 3.2. Coffee Cherry Physicochemical Characterization 

#### 3.2.1. Moisture

Moisture is important because volatile compounds and some carbohydrates can dissolve in coffee bean oil and water [49]. In an evaporating dish (previously dried at 70 °C and weighed), 10 g of coffee were weighed; then, they were collocated in a vacuum stove OV-12 (Jeio Tech, Daejeon, Korea) at 70 °C and −0.05 MPa of pressure. After 24 h, the samples were put into a desiccator for 30 min. Then, the samples were weighed with a Mettler Toledo scale every 24 h until the sample weight was constant.

#### 3.2.2. Dissolved Solids 

Coffee cherry dissolved solids were measured by a digital refractometer (Mettler Toledo, Schwerzenbach, Switzerland). Each measure was made with 3–5 beans, pressing the solids above the equipment lector until they filled the entire interior part. Dissolved solids were used as a ripening and palatability indicator; in addition, in coffee, °Brix reflects the relation between sugars and sensorial characteristics [18].

#### 3.2.3. Color

Coffee color was measured in 10 recently harvested coffee cherry beans. The coffee shell was extracted and set on a white flat surface using a colorimeter CR 14 (Konica Minolta, Tokyo, Japan). The color was reported on an L*a*b* scale. 

#### 3.2.4. pH and Acidity

The pH and acidity of the coffee were measured with Mettler Toledo DL15 titration equipment (Zurich, Switzerland). First, 10–12 g of coffee beans were weighed, and the acids present were extracted by maceration with 50 mL of distilled water for 30 min. The pH was then measured and immediately titrated with 0.01 N NaOH until a pH of 8.2 was reached. The results were converted to mL of NaOH at 0.1 N per 100 g of coffee.

### 3.3. Coffee Fermentation

The dry fermentation process was carried out for 24, 48, 78, and 96 h for each treatment: aerobic dry fermentation, anaerobic dry fermentation, and dry fermentation in an atmosphere modified with CO_2_.

#### 3.3.1. Aerobic Dry Fermentation

This treatment consisted of putting the coffee samples into a bottle half covered, maintaining contact with the air, at environmental conditions of 25 °C and 935.6 HPa of pressure. 

#### 3.3.2. Anaerobic Dry Fermentation

Uncovered bottles were put in a Lab line vacuum chamber (Chicago, IL, USA) at 15 Hg pressure. The air was evacuated by an electric vacuum bomb, which was used to regulate chamber pressure.

#### 3.3.3. Dry Fermentation in an Atmosphere Modified with CO_2_

Bottles were exposed to a CO_2_ gas with the use of a tank; after the exposure, each bottle nozzle contained an air trap coupled to a plastic lid, which was hermetically sealed. Water was added to the air trap to avoid air flow and maintain a modified environment inside the bottle. After taking each sample at the end of the fermentation time, the bottles with the coffee samples were exposed to CO_2_ again. 

### 3.4. Coffee Drying, Threshing, and Roasting 

Each sample, after the fermentation time ended, was dried by an air forced stove at 45 °C until a coffee moisture between 10 and 12% [20] was obtained, weighing samples every 24 h. Then, when the coffee obtained a moisture within the optimal range, samples were threshed with an ING-C-250 thresher (Bogota, Colombia), placed in a container, which was closed with pressure, and threshed to eliminate the mucilage and obtain a green coffee bean. After that, the green coffee beans were roasted with a QuantiK TC 300 A R/G roaster (Quimbaya, Colombia), where the green coffee beans were collocated inside a dumb at 180 °C. During roasting, the temperature was regulated to not exceed 195 °C and not fall below 175 °C. The samples were put into a basket after 12 min of roasting, cooled with a ventilator for 2 min, and re-cooled at an ambient temperature for 10 min. Then, the roasted coffee beans were stored inside Ziploc bags.

### 3.5. Coffee Pulverization

The roasted coffee beans were pulverized by Bunn Coffee Mill equipment (Springfield, IL, USA), which was adjusted to “espresso” mode, pulverizing the coffee into a fine dust. In the equipment hopper, before pulverizing the complete samples, 10–15 beans were added for pre-pulverizing to eliminate the coffee pulverized previously. After this process, the samples were pulverized completely and then stored in Ziploc bags. This pulverized coffee was then ready for extracting and analyzing. 

### 3.6. Volatile Compounds

The volatile compounds in coffee belong to various classes such as acids, aldehydes, esters, furans, alcohols, hydrocarbons, ketones, lactones, phenolic compounds, pyrazines, pyridines, pyrroles, sulfur compounds, and terpenes [50]. Of these compounds, acetic acid, 2-methylpyrazine, furfural or 2-furancarboxaldehyde, 2-furfuryl alcohol or 2-furanmethanol, 2,6-dimethylpyrazine, 5-methylfurfural, and 2-methoxy-4-vinylphenol represented 80% of the relative amount of volatile compounds detected in the coffee samples [26]. Furthermore, these compounds were used to distinguished coffee variety or geographical origin [51,52].

#### 3.6.1. Solid Phase Microextraction (SPME)

The extraction of volatile compounds was carried out using the 50/30 µm PDMS/CAR/DVB fiber supplied by Supelco (57438-U, Bellefonte, PA, USA), as this fiber coating phase allows the extraction of most of the volatile compounds of coffee compared to other fibers such as PDMS, PA, and PDMS/DVB [46,53]. In a 15 mL amber vial, 2 g of ground roasted coffee, 1.785 g of pure NaCl, 5 mL of distilled water at 93 °C, and a 2 mm stirring magnet were added. Subsequently, the vial was kept for 16 min at 93 °C with constant cooling. After this time, the fiber was exposed to the headspace for 35 min, maintaining the temperature and stirring. After extraction, the SPME fiber was immediately inserted into the injection port of the GC–MS for the desorption step at 250 °C for 5 min.

#### 3.6.2. Gas Chromatography–Mass Spectrometry (GC–MS)

The equipment used for compound analysis was a Thermo Fisher Scientific TRACE 1310 gas chromatograph coupled to a Thermo Fisher Scientific ISQ 7000 mass spectrophotometer (San Jose, CA, USA). The GC detector was flame ionization, connected to a TR-5MS column of 5% phenyl polysilphenylene-siloxane phase Thermo Scientific. The initial temperature of the injection from 0 to 5 min was 40 °C; then, from 5 to 51.667 min, the temperature increased from 40 to 180 °C, and in the last phase, from 51.667 to 63.667 min, the temperature increased from 180 to 250 °C. A cooling period began at 65.000 min, when the temperature reached the maximum of 250 °C. Data obtained were analyzed by Chromeleon 7.3 software (Thermo Fisher Scientific, San Jose, CA, USA, 2020), and the retention peaks of each compound were identified within the following ranges:

- MS 60.0–61.0, 42.0–46.0: acetic acid;
- MS 94.0–97.0: 2-methylpyrazine and 2-furancarboxaldehyde;
- MS 98.0–99.0: 2-furanmethanol;
- MS 108.0–109.0: 2,6-dimethylpyrazine;
- MS 110.0–112.0: 5-methylfurfural;
- MS 149.0–151.0: 2-methoxy-4-vinylphenol.

## 4. Conclusions

The aerobic and anaerobic treatments affected the pH, acidity, and concentration of volatile compounds in the coffee during fermentation, because these environments were optimal for the development of the microorganisms responsible for the fermentation process. Moreover, in the aerobic treatment, the concentration of acetic acid doubled after 96 h of fermentation. In contrast, 2-methylpyrazine, 2-furanmethanol, 2,6-dimethylpyrazine, and 5-methylfurfural, decreased their concentrations. 2-furancarboxaldehyde and 2-methoxy-4-vinylphenol maintained their concentrations during fermentation. Furthermore, a decrease in the concentration of all volatile compounds was found in the CO_2_ treatment, due to the inhibition of the development of the microorganisms responsible for fermentation.

## Figures and Tables

**Table 1:** Volatile compound identification.

Compound	TR (min)	KI	KI_R_	Aroma and Taste Description
Acetic acid	3.11 ± 0.04	602.90	605	Acid [27], bitter [28], vinegar [6]
2-methylpyrazine	9.55 ± 0.1	878.18	876	Nut [6,20,29], chocolate [28]
2-furancarboxaldehyde	10.14 ± 0.1	892.89	851	Floral [30]
2-furanmethanol	11.11 ± 0.6	915.31	891	Candy [20], burnt [28]
2,6-dimethylpyrazine	14.20 ± 0.1	972.52	930	Cocoa [20], chocolate [29]
5-methylfurfural	17.01 ± 0.1	1028.15	982	Spicy [20], candy [29]
2-methoxy-4-vinylphenol	34.48 ± 0.04	1390.26	1330	Clove [20], spicy [30]

RT: retention time, KI: Kovats index calculated for the TR-5MS capillary column, KI_R_: Kovats index from the National Institute of Standard Technology (NIST) database: https://webbook.nist.gov/chemistry/name-ser/ (accessed on 20 December 2021).

**Table 2:** Correlation matrix and significance level between variables and compounds.

Variable	Time	pH	Acidity
Time	1	−0.83 ***	0.90 ***
pH	−0.83 ***	1	−0.92 ***
Acidity	0.90 ***	−0.92 ***	1
C1	0.41 **	−0.62 ***	0.59 ***
C2	−0.33 *	0.03 ^ns^	−0.25 ^ns^
C3	−0.20 ^ns^	−0.02 ^ns^	−0.04 ^ns^
C4	−0.40 *	0.16 ^ns^	−0.34 *
C5	−0.49 **	0.24 ^ns^	−0.21 ^ns^
C6	−0.37 *	0.11 ^ns^	−0.14 ^ns^
C7	−0.13 ^ns^	−0.05 ^ns^	0.19 ^ns^

^ns^ Not significant. * Slightly significant. ** Moderately significant. *** Highly significant. C1: Acetic acid. C2: 2-methylpyrazine. C3: 2-furancarboxaldehyde. C4: 2-furanmethanol. C5: 2,6-dimethylpyrazine. C6: 5-methylfurfural. C7: 2-methoxy-4-vinylphenol.

## Data Availability

Data are available from the authors upon reasonable request.

## Sample Availability

Samples of the compounds are available from the authors (J.F. and G.G.).

## References

[1] Abalo R. (2021). Coffee and caffeine consumption for human health. Nutrients.

[2] Seninde D.R., Chambers E. (2020). Coffee flavor: A review. Beverages.

[3] Lee L.W., Cheong M.W., Curran P., Yu B., Liu S.Q. (2015). Coffee fermentation and flavor—An intricate and delicate relationship. Food Chem..

[4] Evangelista S.R., Silva C.F., da Miguel M.G.P.C., de Cordeiro C.S., Pinheiro A.C.M., Duarte W.F., Schwan R.F. (2014). Improvement of coffee beverage quality by using selected yeasts strains during the fermentation in dry process. Food Res. Int..

[5] da Mota M.C.B., Batista N.N., Rabelo M.H.S., Ribeiro D.E., Borém F.M., Schwan R.F. (2020). Influence of fermentation conditions on the sensorial quality of coffee inoculated with yeast. Food Res. Int..

[6] Caporaso N., Whitworth M.B., Cui C., Fisk I.D. (2018). Variability of single bean coffee volatile compounds of Arabica and robusta roasted coffees analysed by SPME-GC-MS. Food Res. Int..

[7] Córdoba Castro N.M., Guerrero Fajardo J.E. (2016). Caracterización de los procesos tradicionales de fermentación de café en el departamento de Nariño. Biotecnol. Sect. Agropecu. Agroind..

[8] Dong W., Hu R., Long Y., Li H., Zhang Y., Zhu K., Chu Z. (2019). Comparative evaluation of the volatile profiles and taste properties of roasted coffee beans as affected by drying method and detected by electronic nose, electronic tongue, and HS-SPME-GC-MS. Food Chem..

[9] Bodner M., Morozova K., Kruathongsri P., Thakeow P., Scampicchio M. (2019). Effect of harvesting altitude, fermentation time and roasting degree on the aroma released by coffee powder monitored by proton transfer reaction mass spectrometry. Eur. Food Res. Technol..

[10] Afriliana A., Pratiwi D., Giyarto G., Belgis M., Harada H., Yushiharu M., Taizo M. (2019). Volatile compounds changes in unfermented robusta coffee by re-fermentation using commercial kefir. Nutr. Food Sci. Int. J..

[11] Yu J.-M., Chu M., Park H., Park J., Lee K.-G. (2021). Analysis of volatile compounds in coffee prepared by various brewing and roasting methods. Foods.

[12] Kim S.J., Lee S., Bang E., Lee S., Rhee J.K., Na Y.C. (2019). Comparative evaluation of flavor compounds in fermented green and roasted coffee beans by solid phase microextraction-gas chromatography/mass spectrometry. Flavour Fragr. J..

[13] de Melo Pereira G.V., de Carvalho Neto D.P., Magalhães Júnior A.I., Vásquez Z.S., Medeiros A.B.P., Vandenberghe L.P.S., Soccol C.R. (2019). Exploring the impacts of postharvest processing on the aroma formation of coffee beans—A review. Food Chem..

[14] de Silva S.A., de Queiroz D.M., de Pinto F.A.C., Santos N.T. (2014). Coffee quality and its relationship with Brix degree and colorimetric information of coffee cherries. Precis. Agric..

[15] Marín-López S.M., Arcila-Pulgarín J., Montoya-Restrepo E.C., Oliveros-Tascón C.E. (2003). Cambios físicos y químicos durante la maduracíon del fruto de café (*Coffea arabica* L. var. Colombia). Cenifcafé.

[16] Carvajal J., Aristizábal I., Oliveros C., Mejía J. (2011). Colorimetría del fruto de café (*Coffea arabica* L.) durante su desarrollo y maduración. Rev. Fac. Nal. Agr. Medellín.

[17] Martins P.M.M., Ribeiro L.S., da Miguel M.G.C.P., Evangelista S.R., Schwan R.F. (2019). Production of coffee (*Coffea arabica*) inoculated with yeasts: Impact on quality. J. Sci. Food Agric..

[18] Silva P.A., Rabelo V.M., Maria Reis Calixto J., De Oliveira Coelho P., Rocha De Carvalho Gorski I. (2014). Quality assessment of coffee grown in Campos Gerais, Minas Gerais State, Brazil. Acta Sci. Technol..

[19] Pothakos V., De Vuyst L., Zhang S.J., De Bruyn F., Verce M., Torres J., Callanan M., Moccand C., Weckx S. (2020). Temporal shotgun metagenomics of an Ecuadorian coffee fermentation process highlights the predominance of lactic acid bacteria. Curr. Res. Biotechnol..

[20] da Silva B.L., Pereira P.V., Bertoli L.D., Silveira D.L., Batista N.N., Pinheiro P.F., de Souza Carneiro J., Schwan R.F., da Silva S.A., Coelho J.M. (2021). Fermentation of *Coffea canephora* inoculated with yeasts: Microbiological, chemical, and sensory characteristics. Food Microbiol..

[21] Farah A. (2012). Coffee constituents. Coffee Emerg. Health Eff. Dis. Prev..

[22] Jackels S.C., Jackels C.F. (2005). Characterization of the coffee mucilage fermentation process using chemical indicators: A field study in Nicaragua. J. Food Sci..

[23] Hameed A., Hussain S.A., Ijaz M.U., Ullah S., Pasha I., Suleria H.A.R. (2018). Farm to consumer: Factors affecting the organoleptic characteristics of coffee. II: Postharvest processing factors. Compr. Rev. Food Sci. Food Saf..

[24] Pereira L.L., Guarçoni R.C., Moreli A.P., Pinheiro P.F., Pinheiro C.A., Moreira T.R., da Siqueira E.A., Ten Caten C.S. (2021). Physicochemical parameters of arabica fermented coffee in different altitudes. Coffee Sci..

[25] Puerta G.I., Ríos-Arias S. (2011). Composición química del mucílago de café, según el tiempo de fermentación y refrigeración. Cenicafé.

[26] Korhoňová M., Hron K., Klimčíková D., Müller L., Bednář P., Barták P. (2009). Coffee aroma—Statistical analysis of compositional data. Talanta.

[27] Vasanthy M., Ravindran B., Chung W.J., Chang S.W. (2021). Treatment of coffee cherry pulping wastewater by using lectin protein isolated from *Ricinus communis* L. seed. J. Water Process Eng..

[28] Yang N., Liu C., Liu X., Degn T.K., Munchow M., Fisk I. (2016). Determination of volatile marker compounds of common coffee roast defects. Food Chem..

[29] Thammarat P., Kulsing C., Wongravee K., Leepipatpiboon N., Nhujak T. (2018). Identification of volatile compounds and selection of discriminant markers for elephant dung coffee using static headspace gas chromatography—Mass spectrometry and chemometrics. Molecules.

[30] Laukaleja I., Kruma Z. (2019). Phenolic and volatile compound composition influence to specialty coffee cup quality. Agron. Res..

[31] Sunarharum W.B., Williams D.J., Smyth H.E. (2014). Complexity of coffee flavor: A compositional and sensory perspective. Food Res. Int..

[32] Schwan R., Silva C., Batista L., Hui Y.H., Evranuz E.Ö. (2012). Coffee Fermentation. Handbook of Plant-Based Fermented Food and Beverage Technology.

[33] Bressani A.P.P., Batista N.N., Ferreira G., Martinez S.J., Simão J.B.P., Dias D.R., Schwan R.F. (2021). Characterization of bioactive, chemical, and sensory compounds from fermented coffees with different yeasts species. Food Res. Int..

[34] Puerta G.I. (2012). Factores, procesos y controles en la fermentacion del cafe. Av. Técn. Cenicafé.

[35] Wamuyu K.A., Richard K., Beatrice M., Cecilia K. (2017). Effect of different fermentation methods on physicochemical composition and sensory quality of coffee (*Coffea arabica*). IOSR J. Environ. Sci. Toxicol. Food Technol..

[36] De Bruyn F., Zhang S.J., Pothakos V., Torres J., Lambot C., Moroni A.V., Callanan M., Sybesma W., Weckx S., De Vuyst L. (2017). Exploring the impacts of postharvest processing on the microbiota and metabolite profiles during green coffee bean production. Appl. Environ. Microbiol..

[37] Puerta G.I. (2010). Fundamentos del proceso de fermentacion en el beneficio de productos como el cafe. Fed. Nac. Cafe. Colomb..

[38] de Carvalho Neto D.P., de Melo Pereira G.V., Finco A.M.O., Letti L.A.J., da Silva B.J.G., Vandenberghe L.P.S., Soccol C.R. (2018). Efficient coffee beans mucilage layer removal using lactic acid fermentation in a stirred-tank bioreactor: Kinetic, metabolic and sensorial studies. Food Biosci..

[39] Haile M., Kang W.H. (2019). The role of microbes in coffee fermentation and their impact on coffee quality. J. Food Qual..

[40] Elhalis H., Cox J., Frank D., Zhao J. (2020). The crucial role of yeasts in the wet fermentation of coffee beans and quality. Int. J. Food Microbiol..

[41] Avallone S., Guyot B., Brillouet J.-M., Olguin E., Guiraud J.-P. (2001). Microbiological and biochemical study of coffee fermentation. Curr. Microbiol..

[42] Chaichi M., Ghasemzadeh-Mohammadi V., Hashemi M., Mohammadi A. (2015). Furanic compounds and furfural in different coffee products by headspace liquid-phase micro-extraction followed by gas chromatography–mass spectrometry: Survey and effect of brewing procedures. Food Addit. Contam. Part B Surveill..

[43] Zapata J., Londoño V., Naranjo M., Osorio J., Lopez C., Quintero M. (2018). Characterization of aroma compounds present in an industrial recovery concentrate of coffee flavour. CyTA J. Food.

[44] Zakidou P., Plati F., Matsakidou A., Varka E.-M., Blekas G., Paraskevopoulou A. (2021). Single origin coffee aroma: From optimized flavor protocols and coffee customization to instrumental volatile characterization and chemometrics. Molecules.

[45] Kipkorir R., Muhoho S., Muliro P., Mugendi B., Frohme M., Broedel O. (2015). Effects of coffee processing technologies on aroma profiles and sensory quality of Ruiru 11 and SL 28 Kenyan coffee varieties. Asian J. Agric. Food Sci..

[46] Angeloni S., Mustafa A.M., Abouelenein D., Alessandroni L., Acquaticci L., Nzekoue F.K., Petrelli R., Sagratini G., Vittori S., Torregiani E. (2021). Characterization of the aroma profile and main key odorants of espresso coffee. Molecules.

[47] Prakash I., Kumar P., Om H., Basavaraj K., Murthy P.S. (2022). Metabolomics and volatile fingerprint of yeast fermented robusta coffee: A value added coffee. LWT.

[48] Yu A.-N., Zhang A.-D. (2010). The effect of pH on the formation of aroma compounds produced by heating a model system containing L-ascorbic acid with L-threonine/L-serine. Food Chem..

[49] Toledo P.R., Pezza L., Pezza H.R., Toci A.T. (2016). Relationship between the different aspects related to coffee quality and their volatile compounds. Compr. Rev. Food Sci. Food Saf..

[50] Cincotta F., Tripodi G., Merlino M., Verzera A., Condurso C. (2020). Variety and shelf-life of coffee packaged in capsules. LWT.

[51] Risticevic S., Carasek E., Pawliszyn J. (2008). Headspace solid-phase microextraction–gas chromatographic–time-of-flight mass spectrometric methodology for geographical origin verification of coffee. Anal. Chim. Acta.

[52] Bicchi C.P., Panero O.M., Pellegrino G.M., Vanni A.C. (1997). Characterization of roasted coffee and coffee beverages by solid phase microextraction−gas chromatography and principal component analysis. J. Agric. Food Chem..

[53] Figueroa J.G., Vargas L.F. (2016). Evaluation of SDE, SFE and SPME/Gc-Ms for extraction and determination of aroma compounds from Vilcabamba-Ecuadorian roasted coffee. Quím. Nova.

